# Effects of Microfiltered Seawater Intake and Variable Resistance Training on Strength, Bone Health, Body Composition, and Quality of Life in Older Women: A 32-Week Randomized, Double-Blinded, Placebo-Controlled Trial

**DOI:** 10.3390/ijerph20064700

**Published:** 2023-03-07

**Authors:** Alvaro Juesas, Pedro Gargallo, Javier Gene-Morales, Carlos Babiloni-López, Angel Saez-Berlanga, Pablo Jiménez-Martínez, Jose Casaña, Josep C. Benitez-Martinez, Rodrigo Ramirez-Campillo, Ivan Chulvi-Medrano, Juan C. Colado

**Affiliations:** 1 Research Group in Prevention and Health in Exercise and Sport (PHES), University of Valencia, 46010 Valencia, Spain; 2 ICEN Institute, 28840 Madrid, Spain; 3 Exercise Intervention for Health Research Group (EXINH-RG), University of Valencia, 46010 Valencia, Spain; 4 Research Group in Physiotherapy Technology and Recovering (FTR), University of Valencia, 46010 Valencia, Spain; 5 Exercise and Rehabilitation Sciences Laboratory, School of Physical Therapy, Faculty of Rehabilitation Sciences, Universidad Andres Bello, Santiago 7591538, Chile; 6 Department of Physical Education and Sports, University of Valencia, 46010, Valencia, Spain

**Keywords:** older adults, postmenopausal, variable resistance training, elastic bands, rate of perceived exertion, musculoskeletal, muscle strength, bone mineral density, body composition, body fat, Short Form Health Survey (SF-36)

## Abstract

The aim was to explore the effects of a 32-week resistance training (RT) intervention with elastic bands with or without microfiltered seawater (SW) supplementation on isokinetic strength, bone mineral density (BMD), body composition, and subjective quality of life in postmenopausal women. Ninety-three untrained women (age: 70.00 ± 6.26 years; body mass index: 22.05 ± 3.20 kg/m^2^; body fat: 37.77 ± 6.38%; 6.66 ± 1.01 s up-and-go test) voluntarily participated in this randomized, double-blinded, controlled trial. Participants were allocated into four groups (RT+SW, RT+PLA, CON+SW, and CON+PLA). The RT intervention (twice weekly) consisted of different exercises for the whole body performed at submaximal intensities with elastic bands. Both control groups were not involved in any exercise program. A two-way mixed analysis of variance of repeated measures revealed significant improvements in almost all the variables in both intervention groups (*p* < 0.05). However, significant differences with controls were encountered in isokinetic strength, body fat percentage, and bodily pain. Although the group with SW supplementation obtained greater effect sizes, non-significant differences between both RT groups were observed. In conclusion, the determinant factor of the adaptations seems to be RT rather than SW.

## 1. Introduction

The management of healthy aging represents an important therapeutic concern for public health and governments [1]. Aging is associated with osteopenia, sarcopenia, overweight, and dynapenia, which increase the risk of functional dependence and reduce the quality of life [2,3]. In this regard, bone health has been proposed as a critical factor in the senescence process due to the reported higher risk of falls, fractures, and mortality in older adults [4]. Previous research has elucidated different procedures for assessing bone quality and health, such as magnetic resonance imaging (MRI), dual-energy, X-ray absorptiometry (DXA), or bone resorption and ossification biomarkers [5,6]. In this sense, although bone mineral density (BMD) assessment by DXA is claimed as the gold standard, this evaluation only provides partial data about bone strength and properties [5]. Thus, biomarkers such as procollagen type I N propeptide (P1NP) and cross-linked C-telopeptides of type I collagen/1000 (BCTX/1000) have been documented as representative measures of bone’s architecture and remodeling rates [7].

Dietary supplements are legal, free-sale nutritional complements that in conjunction with a healthy diet can improve well-being and/or sports performance [8,9]. In this concern, resistance training (RT) and specific nutritional supplementation (e.g., calcium, vitamin D, creatine, and magnesium) have elicited positive results as non-pharmacological strategies to prevent and treat the abovementioned long-term conditions [10,11,12,13,14]. Previous studies have documented the direct benefits of liquid mineral-enriched supplementation, such as seawater (SW) on human health (e.g., immunological and gastrointestinal) [15,16,17] and performance [18,19]. SW has been mostly studied in aerobic-based sports, as it is depicted in a recent systematic review [20]. SW supplementation provokes an ergogenic effect on performance outcomes such as endurance muscle ability [21], incremental running testing [22], and high-intensity intermittent running [19]. Consistently, it has been hypothesized that SW may lower lactate concentrations [22,23] and increase the recovery status after exhaustive endurance tasks [21]. However, despite the promising results of acute endurance exercise, little is known about the chronic effects of SW administration while following a RT program (e.g., weight machines or elastic bands [EB]). In addition, the current knowledge of SW effects in non-athletic populations (e.g., older women) and bone health is scarce.

RT with EB has shown positive acute and chronic adaptations in different population groups, including older women [24,25,26,27,28]. One of the primary concerns during RT bouts is appropriate hydration [29]. In this regard, exhaustive efforts may induce a hypohydration state due to reductions in total body water volume and the increase of extracellular fluid osmolality [30]. As previously mentioned, the intake of water and mineral-enriched supplementation has been reported to restore normal osmolality [31], especially in endurance sports [21]. However, no previous study has investigated the potential beneficial effects that mineral-enriched supplementation, such as SW, before or during RT bouts, may have in long-term adaptations (e.g., body composition, bone health, strength, perceived quality of life).

Therefore, this study aimed to analyze the effects of a mineral-enriched supplement (i.e., microfiltered SW) and 32 weeks of variable RT (i.e., EB) on isokinetic muscle strength (hip adduction, knee flexion, and elbow flexion at 60 and 180°/s), bone health biomarkers (global, hip, and spine bone mineral density, P1NP, BCTX/1000), body composition (fat and muscle mass), and quality of life (SF-36) in older women (>65 years).

It was hypothesized that a 32-week variable (i.e., EB) RT program would increase muscle strength, bone markers, and body composition, with better results when participants were supplemented with SW. Moreover, considering that the participants supplemented with SW would improve the aforementioned parameters, we expected to find an improved subjective quality of life in those participants.

## 2. Materials and Methods

### 2.1. Study Design

This study pertains to a larger research project aimed at exploring the effects of different RT intensities on blood biomarkers and muscular strength. We used a 32-week prospective, randomized, double-blinded, controlled trial design following the Consolidated Standards of Reporting Trials (CONSORT) (Supplementary Materials Table S1). Four study groups were formed (RT+SW, RT+PLA, CON+SW, and CON+PLA). All the participants provided informed consent and were free to withdraw from the study at any time. We applied all procedures following the tenets of the Declaration of Helsinki. The experimental protocols were authorized by the Ethics Committee of the University of Valencia (Valencia, Spain) (H1414072784009). We conducted the procedures in different Municipal Activity Centers for Older People in Valencia (Spain) and measurements in the Sports Performance Laboratory of the Faculty of Physical Activity and Sports Sciences of the University of Valencia (Valencia, Spain) and University Hospital Dr. Peset (Valencia, Spain).

### 2.2. Participants

We recruited participants with an advertisement that was publicly posted at several Municipal Activity Centers for Older People in Valencia (Spain). The inclusion criteria were as follows: (i) women aged ≥ 65 years; (ii) able to climb 10 stairs without pause and walk 100 m without a walker; (iii) score in the mini-mental state examination (MMSE) > 23 points [32]; (iv) less than one hour of physical activity or physical exercise per week throughout the six months before the start of the study. Participants who had suffered any musculoskeletal, cardiovascular, hepatic, renal, pulmonary, neurological, or neuromuscular injury or disorder and/or were taking any type of drug/supplement that may alter the results of the study (e.g., vitamin C, vitamin E, estrogens, beta-blockers, calcitonin, steroid hormones) were excluded.

A total of 160 Caucasian women attended the recruitment calls, of which 51 were discarded. Of these 51 excluded women, 19 refused to participate upon receiving a detailed description of the commitments of the study, and 32 did not meet the inclusion criteria (Parkinson’s disease, *n* = 3; multiple sclerosis, *n* = 2; ongoing treatment with specific medications (diuretic, *n* = 4; hormone replacement therapy, *n* = 5; corticosteroids, *n* = 6); age below 60 years, *n* = 2; score in the mini-mental state examination below 23 points, *n* = 1; engagement in regular strength training, *n* = 2; plans to leave the area during the intervention for a long period, *n* = 1; inability to commit due to scheduling conflicts and time constraints, *n* = 6). Therefore, an independent staff member not involved in the trial or any screening, testing, training procedures, or contact with the participants randomized the 109 women into the four groups, using a computer-generated random permutation procedure.

### 2.3. Procedures

#### 2.3.1. Intervention Protocol

Both control groups (CON+SW, CON+PLA) did not participate in any exercise program. Both intervention groups (RT+SW, RT+PLA) participated in two weekly sessions of 55–60 min on non-consecutive days (separated by 48–72 h) for 32 weeks. Each session was performed in a group, and the individuals always performed the exercises in the same order, alternating between the lower and upper limbs to reduce fatigue [33]. A metronome indicated the speed of execution (2 s each of concentric and eccentric contraction) during the whole session. Likewise, the loads were modified (adapting the color and width of the grip) each week to maintain the appropriate training intensities. Two different intensities were used: (i) high intensity (six submaximal repetitions equivalent to 85% of 1RM); (ii) moderate intensity (15 submaximal repetitions equivalent to 65–70% of 1RM). The level of perceived exertion at the end of each set for both intensities on the OMNI-RES EB scale [34] progressed from 6–7 (“somewhat hard”) in the first four weeks to 8–9 (“hard”) during the last 28 weeks. The participants performed 3 sets per exercise throughout the first 8 weeks, which were increased to 4 for the remaining 24 weeks [35]. Between sets, an active rest (coordination and cognitive tasks) [36] of 120 s was allowed throughout the whole intervention. Between exercises, a 90 s passive rest was allowed throughout the first 16 weeks and the last 8 weeks. From week 17 to week 24, the passive rest time was reduced to 60 s. The participants performed lower and upper extremity exercises during the first 24 weeks. For the last 8 weeks, the exercises were combined in supersets. No pause was allowed between both exercises of the superset. During the first 24 weeks, the participants performed the exercises in the following order: elbow flexion, squat, upright row, lunge, incline row, and standing hip abduction. The order of the supersets for the last 8 weeks was: standing hip abduction + squats, pushups + incline row, and lunges + upright row.

#### 2.3.2. Initial Assessment and Familiarization

The participants completed two familiarization sessions to learn exercise techniques [37] and select the width of the EB grip for each exercise according to prior studies [38]. For such purpose, volunteers performed sets of 6 and 15 repetitions with an EB (Theraband, Hygenic Corporation, Akron, OH, USA; five colors in ascending order of resistance/ thickness: green, blue, black, silver, and gold) at different grip widths. These efforts showed the participants what were low and maximal values (1 to 9) in the OMNI-Resistance exercise scale of perceived exertion with the EB [34]. The bands presented a mark every 3 cm to measure and record the increase or reduction in intensity.

We measured height and body mass with a portable stadiometer (Seca T214, Hamburg, Germany; precision 0.01 cm), and a digital scale (Tanita^®^ BF-350, Tanita Corp., Tokyo, Japan; precision 0.01 kg) following Calatayud et al. [39] protocol. We used DXA (QDR^®^ Hologic Discovery Wi, Hologic Inc., Waltham, MA, USA) equipped with APEX software (version 12.4, APEX Corp., Waltham, MA, USA) to examine body composition (muscle and fat mass), anteroposterior lumbar spine (segments L1–L4), non-dominant proximal femur (total hip), and global bone mineral density. We instructed the participants to control hydration and diet before the DXA measurements to avoid potential influences on the outcomes. The protocol was followed according to Carnevale et al. [40]. The same certified researcher carried out all the measurements.

#### 2.3.3. Supplementation Protocol

The microfiltered SW and placebo supplements used were supplied by Quinton (Laboratories Quinton International, S.L., Alicante, Spain). Participants drank a 20 mL sample just before each session. Composition of this nutritional supplement was as follows: (i) sodium: 11.87 g L^−1^; (ii) chloride: 20.36 g L^−1^; (iii) magnesium: 1.36 g L^−1^; (iv) calcium: 433 mg L^−1^; (v) potassium: 441 mg L^−1^; (vi) bicarbonate: 148 mg L^−1^; (vii) zinc: 11.8 μg L^−1^; (viii): manganese: 116.9 μg L^−1^; (ix) cupper: 6.6 μg L^−1^. Furthermore, the nutritional supplement included other chemical elements: proteins, lipids, water-soluble vitamins D-biotin, thiamine, riboflavin, nicotinamide, cyanocobalamin, pyridoxine, and fat-soluble vitamins retinal, vitamin D3, α-tocopherol and vitamin K1, naturally present in seawater in trace quantities. Placebo composition included only water. This product has neither contraindications nor incompatibilities and does not cause side reactions. A blinded researcher distributed the placebo samples with the same appearance.

### 2.4. Strength Assessment

We used a multi-joint isokinetic dynamometer (Biodex Medical TM, Shirley, NY, USA), with the software Advantage (version 3.2, Biodex System Advantage, Shirley, NY, USA) to measure isokinetic strength [41]. We retrieved maximal strength in hip adduction and knee and elbow flexion at angular speeds of 180 and 60°/s since they are the ideal speeds to verify power/function and maximum force, respectively [42]. The participants performed all three exercises in random order and rested for two minutes between exercises. Two trials (one at each angular velocity) consisting of five maximal voluntary contractions on the dominant side were conducted for each exercise. Each exercise was always evaluated first at an angular velocity of 180°/s, followed by the same exercise at 60°/s. A rest of one minute was allowed between the trial at each angular velocity. We used the best maximum concentric isokinetic torque from the five repetitions for analyses. The knee extension range of movement was from 5 to 90°, the elbow flexion was from 15 to 75°, and the hip adduction was from 5 to 45° [43].

### 2.5. Physiological Parameters

We used serum sample separation to analyze the set of physiological parameters (i.e., P1NP and BCTX/1000). After participants fasted for 12 h, a qualified nurse drew 10 mL whole blood samples from an antecubital vein of the participants in a seated position. Blood samples were extracted into dry 10 mL tubes with a silicone gel separator and coagulation activator between 8:00 and 10:00 a.m. (to minimize circadian effects). These samples were kept in a refrigerator at 2–4°C until they were processed, which always occurred within 4 h of extraction. After clot retraction (15–30 min at room temperature), samples were centrifuged with Histopaque (Sigma H-1077) at 3500 rpm for five minutes at 4°C in a Rotina 380R Hettich centrifuge (Tuttlinger, Germany). The professional in charge pipetted and aliquoted the resulting serum supernatant. The aliquots were frozen at −80°C until use. An automated Roche ECLIA system (Cobas 6000, Roche Diagnostics, Mannheim, Germany) measured serum P1NP and BCTX/1000. The person in charge ran the samples in duplicate as per the manufacturer’s instructions to ensure the reliability of the measurements. If the results differed by more than 15%, the analysis was repeated. We used the average of both readings for data analysis.

### 2.6. Quality of Life Assessment

With the Short Form Health Survey (SF-36) we evaluated physical, psychological, and social well-being. This tool consists of 36 items arranged in eight dimensions that assess positive and negative states of health (general health, physical functioning, physical role, bodily pain, emotional role, social function, vitality, and mental health). For each dimension, the items are coded, aggregated, and transformed into a scale ranging from zero (worst state of health) to 100 (best state of health). A score is achieved for each dimension, as the SF-36 has not been shaped to generate an overall score [44]. Previous research has demonstrated its usefulness and reliability in older adults [45].

### 2.7. Statistical Analyses

We determined the sample size with an a priori analysis conducted with G* Power 3.1 software [46] to reduce the probability of type II error [47]. The calculation based on the study design (F-tests, ANOVA: repeated measures, within–between interaction) indicated a sample size of 72 volunteers to meet a statistical power of 0.80, α = 0.05, a correlation coefficient of 0.5, a non-sphericity correction of 1, and an effect size (ES) of 0.35. We selected the ES according to the average outcomes of all the dependent variables as obtained in the pilot studies.

We used commercial software IBM SPSS (version 26.0; IBM Corp., Armonk, NY, USA) to perform the rest of the analyses based on the principle of the intention to treat. Results are reported as mean and standard deviation (SD). We uniformly set the level of statistical significance at *p* < 0.05.

We checked the normality of data distribution using the Kolmogorov–Smirnov test. We transformed the non-normal variables, first, into a percentile rank and, second, into a normally distributed variable through the inverse normal [48]. Therefore, we carried out a two-way mixed analysis of variance (ANOVA) of repeated measures to determine the influence of each group (RT+SW, RT+PLA, CON+SW, CON+PLA) and time (pre- and post-test) on isokinetic strength, BMD, blood markers, body composition, and quality of life. The eta partial squared (ηp²) served to evaluate the ES, with 0.01 < ηp² < 0.06 constituting a small effect, 0.06 ≤ ηp² ≤ 0.14 a medium effect, and ηp² > 0.14 a large effect. Planned pairwise comparisons were conducted using the Bonferroni post hoc correction to test for differences. We used Cohen’s d to calculate the ES of the post hoc comparisons, which was interpreted as a trivial (<0.20), small (0.20–0.49), moderate (0.50–0.79), or large effect (≥0.80) [49].

## 3. Results

### 3.1. Participants

**Figure 1:**
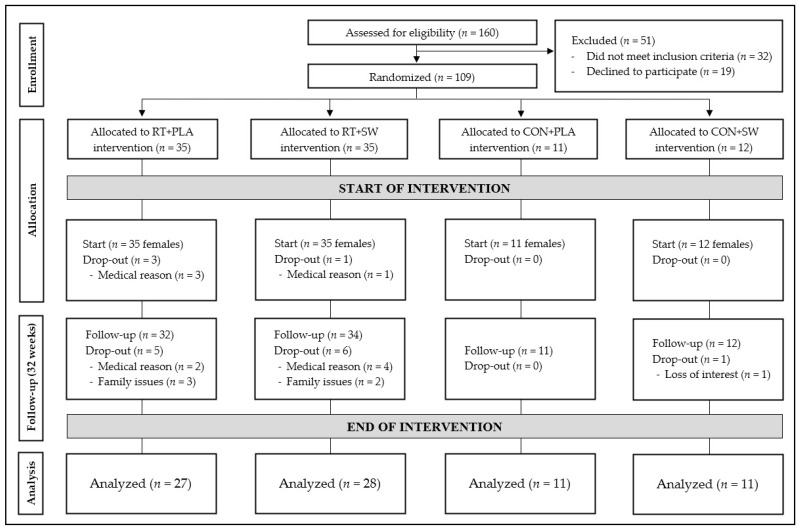
Flowchart of participation. RT: resistance training; PLA: placebo; SW: microfiltered seawater; CON: control group.

Details of the participant flow through the study are displayed in Figure 1.

Ninety-three untrained older women were randomly assigned into four groups: (i) resistance training with deep seawater supplementation (RT+SW; n = 35); (ii) resistance training with placebo supplementation (RT+PLA; n = 35); (iii) control group (no exercise) with deep seawater supplementation (CON+SW; n = 11); (iv) control group (no exercise) with placebo supplementation (CON+PLA, n = 12). The baseline characteristics of the subjects are presented in Table 1. At baseline, the age, anthropometric characteristics, and TUG performance did not differ between the intervention groups (*p* > 0.05, ηp^2^ < 0.06).

Of the 109 women definitively randomized into the four groups, 93 started the intervention and 77 completed the 32-week intervention (dropout rate of 17.2%). At the end of the training program (Week 32), the attendance rate was approximately 75%.

### 3.2. Strength

The ANOVA testing (see Table 2) showed a significant effect of time on knee and elbow flexion at both speeds. Additionally, the interaction group x time showed a significant effect on all the isokinetic strength variables.

Table 3 presents descriptive and inferential analyses performed on the isokinetic strength variables. While both resistance training groups significantly improved all the variables (greater ES for RT+SW), both control groups presented non-significant variations. The post hoc between-group comparison (Supplementary Materials Table S2) revealed non-significant differences between RT+SW and RT+PLA. On the other hand, both resistance training groups presented significantly greater levels of post-intervention isokinetic strength levels compared to both control groups.

### 3.3. Bone Health

Table 4 shows the results of the ANOVA performed on the bone health parameters assessed. A significant effect of time was observed on hip BMD and P1NP. Furthermore, the interaction group x time showed significant effects on all the bone health parameters.

The post hoc between-group comparisons (Supplementary Materials Table S3) showed non-significant differences between groups. As can be seen in Table 5, while RT+SW significantly improved all the variables, RT+PLA only improved hip BMD, P1NP, and BCTX/1000, and both control groups presented non-significant variations in all the bone markers.

### 3.4. Body Composition

Table 6 depicts the effects of the factor time and the interaction group x time on body composition. It is worth mentioning that only muscle mass and fat percentage were influenced by both factors.

Table 7 presents descriptive and inferential analyses performed on body composition parameters. While RT+SW significantly improved all the variables, RT+PLA only improved muscle mass and body fat percentage, and both control groups presented non-significant variations in body composition. The post hoc between-group analysis (Supplementary Materials Table S4) revealed non-significant differences between the study groups apart from those presented in the table.

### 3.5. Quality of Life

The ANOVA performed on SF-36 (Table 8) showed a significant effect of time on general health, bodily pain, emotional role, vitality, and mental health. On the other hand, the interaction group x time only showed significant effects on vitality.

Table 9 exhibits the eight dimensions covered by the SF-36 questionnaire. While both resistance training groups significantly improved almost all the variables (greater ES for RT+SW), both control groups presented non-significant variations. The post hoc between-group comparison (Supplementary Materials Table S5) showed non-significant between-group differences apart from those presented in the table.

## 4. Discussion

This study aimed to explore the effects of a mineral-enriched supplement and 32 weeks of a RT intervention with EB on isokinetic muscle strength (hip adduction, knee flexion, and elbow flexion at 60 and 180°/s), bone markers (global BMD, hip BMD, spine BMD, P1NP, and BCTX/1000), body composition (fat and muscle mass), and quality of life (SF-36). The main finding of the present study was that a RT program with EB and SW supplementation over 32 weeks improved all the analyzed parameters of strength, bone health, body composition, and almost all the quality-of-life parameters. While non-significant between-group differences existed in the baseline measurements, significant differences in the post-intervention measurements were observed between both RT groups and control groups in isokinetic strength, body fat percentage, and bodily pain. Non-significant differences existed in post-test measures between both intervention groups (RT+SW vs. RT+PLA), although the RT+SW group presented greater ES. Furthermore, the RT+PLA group did not improve hip adduction strength at 180°/s, global and spine BMD, fat mass, subjective physical functioning, and physical role. Considering the non-significant between-group differences and that the RT+SW group improved all the aforementioned parameters, we could not confirm the study hypothesis.

### 4.1. Strength Adaptations

Long-term RT programs increase lower and upper limb strength in older adults [50,51]. However, the loss of essential minerals (Na, Ca, K, and Mg) caused by fluid depletion during physical exercise may hinder RT performance [52,53]. The intake of SW before RT showed greater ES in almost all the variables of isokinetic strength compared to not drinking SW before RT, although non-significant between-group differences existed. Thus, the ingestion of SW may be able to counteract exercise-induced muscle damage and reinforce the antioxidant ability against oxidative stress [53,54]. The non-significant variations of the CON+SW group are in accordance with previous long-term studies that used mineral supplementation (i.e., magnesium) without a RT intervention and did not show significant effects on isokinetic muscle strength [55,56]. Additionally, calcium supplementation added to RT did not elicit a better improvement in isokinetic leg flexion and extension [57]. Therefore, our study reinforces the hypothesis that RT may be plausibly the determining factor in the development of lower and upper limb strength [58,59]. In this sense, according to previous research, EB training is a safe, portable, effective, progressive overload methodology that can be used everywhere and at any time for increasing muscle mass and strength [27,60].

### 4.2. Bone Health

Regarding bone health, postmenopausal women express an upper bone turnover and a higher rate of trabecular bone loss, mainly in the vertebrae, caused by estrogen deficiency after menopause [61]. Indeed, bone resorption in this population increases by up to 90%, while bone formation increases only up to 45%, as analyzed previously by markers of resorption (BCTX/1000) and bone formation (P1NP) [61]. In our study, non-significant differences appeared between the study groups. However, we found a significant increase in bone formation (P1NP) and a significant decrease in bone resorption (BCTX/1000) in both intervention groups (RT+SW and RT+PLA). Regarding BMD, the intake of SW in the RT+SW group elicited a significant improvement in global BMD, hip BMD, and spine BMD. The intervention group that did not take the supplement (RT+PLA) only reached significant improvements in hip BMD.

Within this context, previous research has detailed the role of magnesium in the prevention and treatment of osteoporosis. Rude et al. [62] reported an increment of 140% of interleukin-1 (IL-1) in comparison to a placebo condition after three days of magnesium depletion in rats’ osteoclasts. Moreover, SW administration in rats led to a rise in osteogenesis rates due to the upregulation in osteoblast differentiation [63]. Orchard et al. [64] reported a relationship between a low magnesium intake and the reduction of hip and total body BMD in postmenopausal older women. In this sense, participants with intakes below 206.5 mg/day showed a 3% reduction in magnesium levels compared to participants with intakes higher than 422.5 mg/day.

Aligned with previous literature [65,66], we found a moderate positive effect of the RT intervention on bone resorption, although significant between-group differences did not exist. In this regard, the impact of RT on BMD has been categorized according to different intensities of the 1RM in postmenopausal women [67]. In this sense, moderate and high-intensity zones (i.e., >70% RM) exhibited greater benefits on bone health and BMD. For this reason, the results found in this study for global, hip, and spine BMD may be explained by the selected intensity in both intervention groups.

### 4.3. Body Composition

Abdominal obesity and low muscular strength levels are linked to a higher risk of hospitalization and dependence [68]. In this sense, RT is crucial to prevent and revert frailty in community-dwelling older people [69]. While the intake of mineral-enriched supplements alone has not shown a significant impact on body composition [55,70], RT is a crucial variable for that purpose [71]. In our study, non-significant differences appeared between the study groups. However, both RT groups presented significant improvements in body composition, except the RT+PLA group, which did not improve fat mass (kg). A plausible explanation for the small ES encountered could be the lower concentrations of magnesium and calcium in SW (magnesium: 26 mg; calcium: 8 mg) compared to other studies (magnesium: 250 mg; calcium: 1200 mg) [55,70].

### 4.4. Quality of Life

Improving the quality of life is the cornerstone in most older adult interventions [72]. Almost all SF-36 questionnaire parameters improved in both RT groups with greater ES for the RT+SW group. However, significant between-group differences only appeared in bodily pain. Concerning the relationship between quality of life and supplement intake, the current literature is ambiguous and depends on the active principle analyzed [73,74,75,76]. In this regard, RT is a major predictor for improving the quality of life in older adults [77]. However, although the relationship between biopsychosocial factors that influence pain is widely studied [78], further studies are needed to elucidate the exact influence of SW on chronic pain in the dimensions of the SF-36 questionnaire retrieved.

### 4.5. Limitations

Despite the novel findings presented, the methodology carried out in this study does not show measurements of the acute effects that SW could have on specific parameters of RT. Whether variables such as levels of acute fatigue and one-repetition maximum vary when participants drink SW just before or during RT is still unknown. Therefore, further investigation of this mineral-enriched supplement is needed.

## 5. Conclusions

An EB RT program with SW supplementation significantly improves isokinetic strength compared to controls. Similar results were obtained in the RT+PLA group. Although both EB RT groups improved bone health, body composition, and quality of life, non-significant differences existed compared to control groups. Only significantly improved bodily pain was found in the RT+SW group compared to controls. Therefore, SW could be used in combination with RT in healthy Caucasian older women without affecting the studied variables.

## Figures and Tables

**Table 1:** Baseline descriptive characteristics of the participants.

	1 RT+PLA (n = 35)	2 RT+SW (n = 35)	3 CON+PLA (n = 12)	4 CON+SW (n = 11)
Age	69.17 ± 5.71	70.80 ± 5.86	67.90 ± 8.60	72.00 ± 7.07
Height (cm)	152.63 ± 4.54	153.46 ± 5.97	150.95 ± 5.19	153.24 ± 5.35
Weight (kg)	66.25 ± 9.41	68.25 ± 11.41	64.36 ± 6.80	71.67 ± 10.46
BMI (kg/m^2^)	28.41 ± 3.99	28.83 ± 4.73	28.50 ± 3.57	29.88 ± 4.69
Fat mass (%)	43.15 ± 1.39	43.94 ± 4.56	41.66 ± 3.26	45.91 ± 5.43
UGT (seconds)	6.74 ± 1.00	6.79 ± 0.86	6.39 ± 1.61	6.38 ± 0.87

BMI: body mass index; UGT: up-and-go test. RT: resistance training; PLA: placebo; SW: microfiltered seawater; CON: control group.

**Table 2:** Results of the ANOVA on isokinetic neuromuscular strength.

	Time			Group x Time		
	F	*p*	ηp^2^	F	*p*	ηp^2^
Hip adduction 60°/s	3.043	0.085	0.034	10.355	<0.001	0.265
Hip adduction 180°/s	0.889	0.348	0.010	4.504	0.006	0.136
Knee flexion 60°/s	4.204	0.043	0.047	5.717	0.001	0.166
Knee flexion 180°/s	8.713	0.004	0.092	5.291	0.002	0.156
Elbow flexion 60°/s	6.462	0.013	0.070	2.639	0.050	0.084
Elbow flexion 180°/s	4.790	0.031	0.053	6.435	<0.001	0.183

F: ANOVA statistic; *p*: *p*-value of significance; ηp^2^: partial eta squared as a measure of the effect size.

**Table 3:** Isokinetic neuromuscular strength.

	1 RT+PLA (n = 35)		2 RT+SW (n = 35)		3 CON+PLA (n = 12)		4 CON+SW (n = 11)	
	Pre	Post	Pre	Post	Pre	Post	Pre	Post
Hip adduction 60°/s (N·m)	42.58 ± 10.81	46.43 ± 12.76 *	38.24 ± 13.40	50.95 ± 16.27 *^,3^	38.30 ± 24.79	35.78 ± 21.51	40.82 ± 19.68	36.24 ± 21.06
Δ	3.61	*p* = 0.04; d = 0.36	12.34	*p* < 0.001; d = 0.85	−2.52	*p* > 0.05	−4.57	*p* > 0.05
Hip adduction 180°/s (N·m)	52.48 ± 18.54	52.65 ± 19.96	40.93 ± 17.52	51.48 ± 20.32 *	41.23 ± 30.74	40.15 ± 25.90	37.94 ± 17.64	34.36 ± 16.94
Δ	0.24	*p* > 0.05	10.24	*p* < 0.001; d = 0.56	−1.08	*p* > 0.05	−3.57	*p* > 0.05
Knee flexion 60°/s (N·m)	40.77 ± 9.21	45.25 ± 13.37 *	41.77 ± 11.28	52.60 ± 14.29 *^,3,4^	39.13 ± 14.38	36.82 ± 11.92	36.17 ± 6.19	34.24 ± 8.75
Δ	4.40	*p* = 0.03; d = 0.39	10.52	*p* < 0.001; d = 0.84	−2.31	*p* > 0.05	−1.93	*p* > 0.05
Knee flexion 180°/s (N·m)	37.83 ± 8.08	42.90 ± 10.47 *^,3,4^	34.63 ± 10.66	43.99 ± 11.62 *^,3,4^	31.69 ± 9.79	30.55 ± 10.99	30.79 ± 8.64	30.66 ± 9.08
Δ	5.06	*p* = 0.002; d = 0.54	9.09	*p* < 0.001; d = 0.84	−1.14	*p* > 0.05	−0.13	*p* > 0.05
Elbow flexion 60°/s (N·m)	16.77 ± 4.05	20.99 ± 5.03 *^,3^	16.18 ± 6.75	21.02 ± 6.97 *^,3^	14.90 ± 7.89	13.96 ± 6.18	16.34 ± 4.11	16.45 ± 5.33
Δ	4.40	*p* < 0.001; d = 0.92	10.52	*p* < 0.001; d = 0.70	−2.31	*p* > 0.05	−1.93	*p* > 0.05
Elbow flexion 180°/s (N·m)	16.14 ± 3.93	20.32 ± 7.76 *^,3^	14.86 ± 5.71	21.34 ± 6.87 *^,3^	13.17 ± 4.80	12.52 ± 4.78	17.40 ± 6.91	15.29 ± 5.83
Δ	4.02	*p* = 0.001; d = 0.68	6.29	*p* < 0.001; d = 1.03	−0.65	*p* > 0.05	−2.11	*p* > 0.05

* Significant differences (*p* < 0.05) between pre- and post-measurements. ^1–4^ Significant differences (*p* < 0.05) with groups 1, 2, 3, or 4, respectively. RT: resistance training; PLA: placebo; SW: microfiltered seawater; CON: control group; N: newton; m: meter; Δ: post-measurement minus pre-measurement; *p*: *p*-value of significance; d: Cohen’s d as a measure of the effect size.

**Table 4:** Results of the ANOVA on bone markers.

	Time			Group x Time		
	F	*p*	ηp^2^	F	*p*	ηp^2^
Global BMD	1.389	0.242	0.016	3.794	0.013	0.117
Hip BMD	4.721	0.033	0.052	4.338	0.007	0.131
Spine BMD	0.021	0.886	0.000	4.492	0.006	0.135
P1NP	8.999	0.004	0.095	4.232	0.008	0.129
BCTX/1000	0.826	0.366	0.010	4.444	0.006	0.134

F: ANOVA statistic; *p*: *p*-value of significance; ηp^2^: partial eta squared as a measure of the effect size.

**Table 5:** Bone markers.

	1 RT+PLA (n = 35)		2 RT+SW (n = 35)		3 CON+PLA (n = 12)		4 CON+SW (n = 11)	
	Pre	Post	Pre	Post	Pre	Post	Pre	Post
Global BMD (g/cm^2^)	0.99 ± 0.10	1.00 ± 0.10	1.03 ± 0.84	1.04 ± 0.09 *	1.02 ± 0.10	1.02 ± 0.10	1.01 ± 0.11	1.00 ± 0.11
Δ	0.01	*p* > 0.05	0.02	*p* < 0.001; d = 0.1	0.00	*p* > 0.05	−0.01	*p* > 0.05
Hip BMD (g/cm^2^)	0.82 ± 0.11	0.83 ± 0.11 *	0.85 ± 0.10	0.87 ± 0.11 *	0.84 ± 0.12	0.83 ± 0.11	0.85 ± 0.14	0.85 ± 0.13
Δ	0.01	*p* = 0.005; d = 0.09	0.01	*p* < 0.001; d = 0.19	0.00	*p* > 0.05	0.00	*p* > 0.05
Spine BMD (g/cm^2^)	0.84 ± 0.13	0.84 ± 0.13	0.87 ± 0.13	0.88 ± 0.13 *	0.82 ± 0.09	0.81 ± 0.08	0.89 ± 0.16	0.88 ± 0.15
Δ	0.01	*p* > 0.05	0.01	*p* < 0.001; d = 0.08	−0.01	*p* > 0.05	0.00	*p* > 0.05
P1NP (μg/L)	35.98 ± 12.17	43.38 ± 10.88 *	34.09 ± 12.92	42.36 ± 11.28 *	43.24 ± 19.94	41.44 ± 16.73	39.35 ± 12.76	40.25 ± 13.28
Δ	7.14	*p* < 0.001; d = 0.64	8.03	*p* < 0.001; d = 0.68	−1.80	*p* > 0.05	0.90	*p* > 0.05
BCTX/1000 (pg/mL)	0.30 ± 0.14	0.28 ± 0.12 *	0.30 ± 0.12	0.27 ± 0.12 *	0.31 ± 0.12	0.32 ± 0.12	0.36 ± 0.13	0.38 ± 0.13
Δ	−0.02	*p* = 0.01; d = 0.15	−0.03	*p* < 0.001; d = 0.25	0.01	*p* > 0.05	0.02	*p* > 0.05

* Significant differences (*p* < 0.05) between pre- and post-measurements. ^1–4^ Significant differences (*p* < 0.05) with groups 1, 2, 3, or 4, respectively. RT: resistance training; PLA: placebo; SW: microfiltered seawater; CON: control group; BMD: bone mineral density; P1NP: procollagen type 1 N-terminal propeptide; BCTX/1000: beta C-terminal telopeptide/1000; g: grams; cm: centimeters; μg/L: microgram per liter; pg/mL: picograms per milliliter; Δ: post-measurement minus pre-measurement; *p*: *p*-value of significance; d: Cohen’s d as a measure of the effect size.

**Table 6:** Results of the ANOVA on body composition.

	Time			Group x Time		
	F	*p*	ηp^2^	F	*p*	ηp^2^
Fat mass (kg)	2.147	0.146	0.024	2.489	0.066	0.078
Muscle mass (kg)	10.027	0.002	0.102	6.517	<0.001	0.182
% Fat mass	7.974	0.006	0.083	6.247	<0.001	0.176

F: ANOVA statistic; *p*: *p*-value of significance; ηp^2^: partial eta squared as a measure of the effect size.

**Table 7:** Body composition.

	1 RT+PLA (n = 35)		2 RT+SW (n = 35)		3 CON+PLA (n = 12)		4 CON+SW (n = 11)	
	Pre	Post	Pre	Post	Pre	Post	Pre	Post
Fat mass (kg)	28.32 ± 6.46	27.30 ± 6.09	30.32 ± 6.35	28.13 ± 7.43 *	26.58 ± 45.95	26.96 ± 4.70	32.30 ± 6.68	32.65 ± 6.70
Δ	−1.02	*p* > 0.05	−2.19	*p* < 0.001; d = 0.32	0.37	*p* > 0.05	0.36	*p* > 0.05
Muscle mass (kg)	35.78 ± 3.90	36.79 ± 4.21 *	36.35 ± 4.69	37.12 ± 4.74 *	35.10 ± 2.81	34.80 ± 2.64	35.90 ± 5.43	35.95 ± 5.55
Δ	1.019	*p* < 0.001; d = 0.25	0.76	*p* < 0.001; d = 0.16	−0.30	*p* > 0.05	0.04	*p* > 0.05
% Fat mass	43.15 ± 1.39	42.11 ± 4.57 *^,4^	43.94 ± 4.56	42.79 ± 4.46 *	41.66 ± 3.26	41.62 ± 3.34	45.91 ± 5.43	46.37 ± 5.16
Δ	−1.04	*p* < 0.001; d = 0.31	−1.15	*p* < 0.001; d = 0.25	−0.04	*p* > 0.05	0.46	*p* > 0.05

* Significant differences (*p* < 0.05) between pre- and post-measurements. ^1–4^ Significant differences (*p* < 0.05) with groups 1, 2, 3, or 4, respectively. RT: resistance training; PLA: placebo; SW: microfiltered seawater; CON: control group; kg: kilograms; Δ: post-measurement minus pre-measurement; *p*: *p*-value of significance; d: Cohen’s d as a measure of the effect size.

**Table 8:** Results of the ANOVA on SF-36.

	Time			Group x Time		
	F	*p*	ηp^2^	F	*p*	ηp^2^
General health	13.621	<0.001	0.135	0.592	0.622	0.020
Physical functioning	0.721	0.398	0.008	2.632	0.055	0.083
Physical role	0.888	0.349	0.010	0.329	0.805	0.011
Bodily pain	8.547	0.004	0.089	1.956	0.127	0.063
Emotional role	4.001	0.049	0.044	0.505	0.680	0.017
Social function	3.124	0.081	0.035	1.050	0.375	0.035
Vitality	18.329	<0.001	0.174	3.893	0.012	0.118
Mental health	7.699	0.007	0.081	1.223	0.306	0.040

F: ANOVA statistic; *p*: *p*-value of significance; ηp^2^: partial eta squared as a measure of the effect size.

**Table 9:** SF-36 questionnaire.

	1 RT+PLA (n = 35)		2 RT+SW (n = 35)		3 CG+Placebo (n = 12)		4 CG+SW (n = 11)	
	Pre	Post	Pre	Post	Pre	Post	Pre	Post
General health	60.17 ± 15.79	70.69 ± 13.89 *	63.53 ± 16.72	75.42 ± 17.35 *	67.00 ± 21.50	70.70 ± 14.43	62.73 ± 16.33	70.29 ± 9.44
Δ	4.02	*p* = 0.001; d = 0.71	6.29	*p* < 0.001; d = 0.70	−0.65	*p* > 0.05	−2.11	*p* > 0.05
Physical functioning	78.57 ± 19.80	82.53 ± 16.27	76.21 ± 17.08	85.01 ± 14.90 *	75.00 ± 28.09	72.15 ± 23.93	76.36 ± 11.20	72.15 ± 23.93
Δ	4.09	*p* > 0.05	4.71	*p* = 0.003; d = 0.55	−0.94	*p* > 0.05	0.12	*p* > 0.05
Physical role	88.74 ± 20.20	91.21 ± 21.19	94.74 ± 11.46	95.37 ± 10.29	89.04 ± 23.90	95.86 ± 8.08	87.76 ± 25.63	85.65 ± 22.09
Δ	2.43	*p* > 0.05	2.55	*p* > 0.05	6.86	*p* > 0.05	−2.08	*p* > 0.05
Bodily pain	65.29 ± 22.22	72.41 ± 17.77 *	69.48 ± 19.66	84.02 ± 18.21 *^,3^	62.75 ± 28.85	64.61 ± 27.40	69.77 ± 18.28	74.03 ± 9.46
Δ	6.09	*p* = 0.03; d = 0.35	15.29	*p* < 0.001; d = 0.77	1.86	*p* > 0.05	4.26	*p* > 0.05
Emotional role	88.26 ± 22.15	96.23 ± 12.31 *	93.08 ± 17.23	98.51 ± 5.73	90.00 ± 22.50	99.71 ± 0.92	93.13 ± 20.02	92.62 ± 19.73
Δ	7.74	*p* = 0.04; d = 0.44	5.19	*p* > 0.05	9.71	*p* > 0.05	−0.52	*p* > 0.05
Social function	81.07 ± 22.15	88.06 ± 17.58 *	84.93 ± 17.62	94.02 ± 9.04 *	96.25 ± 8.44	92.86 ± 10.41	87.50 ± 19.36	92.23 ± 11.01
Δ	6.17	*p* = 0.03; d = 0.35	8.59	*p* = 0.01; d = 0.65	−3.39	*p* > 0.05	4.73	*p* > 0.05
Vitality	61.52 ± 16.98	73.75 ± 15.79 *	60.80 ± 17.13	77.51 ± 16.36 *	63.02 ± 24.78	64.05 ± 19.79	63.32 ± 19.12	66.20 ± 13.35
Δ	12.23	*p* < 0.001; d = 0.75	16.11	*p* < 0.001; d = 1.00	1.03	*p* > 0.05	2.88	*p* > 0.05
Mental health	66.37 ± 19.21	75.01 ± 17.70 *	70.35 ± 16.23	80.35 ± 12.67 *	73.20 ± 12.11	74.58 ± 13.02	74.18 ± 22.41	76.30 ± 13.74
Δ	9.70	*p* = 0.002; d = 0.47	9.82	*p* < 0.001; d = 0.69	1.38	*p* > 0.05	2.12	*p* > 0.05

* Significant differences (*p* < 0.05) between pre- and post-measurements. ^1–4^ Significant differences (*p* < 0.05) with groups 1, 2, 3, or 4, respectively. RT: resistance training; PLA: placebo; SW: microfiltered seawater; CON: control group; Δ: post-measurement minus pre-measurement; *p*: *p*-value of significance; d: Cohen’s d as a measure of the effect size.

## Data Availability

All data generated or analysed during this study are included in this published article and the Supplementary Material. The databases are available upon reasonable request to the corresponding author.

## Supplementary Materials

The following supporting information can be downloaded at: https://www.mdpi.com/article/10.3390/ijerph20064700/s1, Table S1: CONSORT 2010 checklist of information to include when reporting a randomised trial*; Table S2: Post-hoc test on isokinetic neuromuscular strength.; Table S3: Post-hoc test on bone markers.; Table S4: Post-hoc test on body composition.; Table S5: Post-hoc test on SF-36.
