# Supplementary material for: Effects of Microfiltered Seawater Intake and Variable Resistance Training on Strength, Bone Health, Body Composition, and Quality of Life in Older Women: A 32-Week Randomized, Double-Blinded, Placebo-Controlled Trial
Source: Int J Environ Res Public Health. 2023 Mar 7;20(6):4700. doi: 10.3390/ijerph20064700 (PMC10048547; doi:10.3390/ijerph20064700)
Supplement: Supplementary file 1 [file ijerph-20-04700-s001.zip › ijerph-2186559-supplementary.pdf]

CONSORT 2010 checklist of information to include when reporting a randomised trial* (Supplementary Materials Table S1)

Section/Topic	Item No	Checklist item	Reported on page No
**Title and abstract**			
	1a	Identification as a randomised trial in the title	1 (lines 4-5)
	1b	Structured summary of trial design, methods, results, and conclusions (for specific guidance see CONSORT for abstracts)	1 (lines 18-34)
**Introduction**			
Background and objectives	2a	Scientific background and explanation of rationale	1-2 (lines 37-76)
	2b	Specific objectives or hypotheses	2 (lines 77-86)
**Methods**			
Trial design	3a	Description of trial design (such as parallel, factorial) including allocation ratio	3 (lines 90-93)
	3b	Important changes to methods after trial commencement (such as eligibility criteria), with reasons	N.A.
Participants	4a	Eligibility criteria for participants	3 (lines 102-111)
	4b	Settings and locations where the data were collected	3 (lines 285-287)
Interventions	5	The interventions for each group with sufficient details to allow replication, including how and when they were actually administered	3-4 (lines 126-181
Outcomes	6a	Completely defined pre-specified primary and secondary outcome measures, including how and when they were assessed	4-5 (lines 183-220)
	6b	Any changes to trial outcomes after the trial commenced, with reasons	N.A.
Sample size	7a	How sample size was determined	5 (lines 222-226)
	7b	When applicable, explanation of any interim analyses and stopping guidelines	N.A.
Randomisation:			
Sequence	8a	Method used to generate the random allocation sequence	3 (lines 120-

generation			123)
	8b	Type of randomisation; details of any restriction (such as blocking and block size)	3 (lines 120-123)
Allocation concealment mechanism	9	Mechanism used to implement the random allocation sequence (such as sequentially numbered containers), describing any steps taken to conceal the sequence until interventions were assigned	3 (lines 120-123)
Implementation	10	Who generated the random allocation sequence, who enrolled participants, and who assigned participants to interventions	3 (lines 120-123)
Blinding	11a	If done, who was blinded after assignment to interventions (for example, participants, care providers, those assessing outcomes) and how	4 (lines 180-181)
	11b	If relevant, description of the similarity of interventions	N.A.
Statistical methods	12a	Statistical methods used to compare groups for primary and secondary outcomes	5 (lines 233-238)
	12b	Methods for additional analyses, such as subgroup analyses and adjusted analyses	5 (lines 239-244)
**Results**			
Participant flow (a diagram is strongly recommended)	13a	For each group, the numbers of participants who were randomly assigned, received intended treatment, and were analysed for the primary outcome	6 (line 248)
	13b	For each group, losses and exclusions after randomisation, together with reasons	6 (line 248)
Recruitment	14a	Dates defining the periods of recruitment and follow-up	N.A.
	14b	Why the trial ended or was stopped	3 (lines 112-120)
Baseline data	15	A table showing baseline demographic and clinical characteristics for each group	7 (line 1065)
Numbers analysed	16	For each group, number of participants (denominator) included in each analysis and whether the analysis was by original assigned groups	6 (lines 997-1003)
Outcomes and estimation	17a	For each primary and secondary outcome, results for each group, and the estimated effect size and its precision (such as 95% confidence interval)	7 (line 1072)<br>8 (line 1209)<br>8 (line 1219)<br>8 (line 1226)<br>9 (line 1286)<br>9 (line 1295)<br>10 (line 1343)<br>10 (line 1351)

			(Also in supplementary files)
	17b	For binary outcomes, presentation of both absolute and relative effect sizes is recommended	N.A.
Ancillary analyses	18	Results of any other analyses performed, including subgroup analyses and adjusted analyses, distinguishing pre-specified from exploratory	N.A.
Harms	19	All important harms or unintended effects in each group (for specific guidance see CONSORT for harms)	N.A.
**Discussion**			
Limitations	20	Trial limitations, addressing sources of potential bias, imprecision, and, if relevant, multiplicity of analyses	12-13 (lines 452-456)
Generalisability	21	Generalisability (external validity, applicability) of the trial findings	11 (lines 1398-1412) 13 (lines 1858-1864)
Interpretation	22	Interpretation consistent with results, balancing benefits and harms, and considering other relevant evidence	11-13 (lines 1415-1856)
**Other information**			
Registration	23	Registration number and name of trial registry	3 (lines 283-284)
Protocol	24	Where the full trial protocol can be accessed, if available	13 (lines 1879-1881)
Funding	25	Sources of funding and other support (such as supply of drugs), role of funders	13 (lines 1870-1873)

*We strongly recommend reading this statement in conjunction with the CONSORT 2010 Explanation and Elaboration for important clarifications on all the items. If relevant, we also recommend reading CONSORT extensions for cluster randomised trials, non-inferiority and equivalence trials, non-pharmacological treatments, herbal interventions, and pragmatic trials. Additional extensions are forthcoming: for those and for up to date references relevant to this checklist, see www.consort-statement.org.

Supplementary Materials Table S2. Post-hoc test on isokinetic neuromuscular strength.

Measure	Groups being compared		Mean Difference	Sig.	95% Confidence Interval	
					Lower Bound	Upper Bound
Hip adduction 180º/s	RT+PLA	RT+SW	4.67	.66	-6.04	15.38
		CON+PLA	17.90*	.03	1.15	34.64
		CON+SW	14.53	.07	-.96	30.01
	RT+SW	RT+PLA	-4.67	.66	-15.38	6.04
		CON+PLA	13.23	.17	-3.51	29.97
		CON+SW	9.86	.35	-5.63	25.35
	CON+PLA	RT+PLA	-17.90*	.03	-34.64	-1.15
		RT+SW	-13.23	.17	-29.97	3.51
		CON+SW	-3.37	.97	-23.51	16.77
	CON+SW	RT+PLA	-14.53	.07	-30.01	.96
		RT+SW	-9.86	.35	-25.35	5.63
		CON+PLA	3.37	.97	-16.77	23.51
Hip adduction 60º/s	RT+PLA	RT+SW	.54	1.00	-8.43	9.50
		CON+PLA	12.01	.12	-2.01	26.03
		CON+SW	6.64	.54	-6.32	19.61
	RT+SW	RT+PLA	-.54	1.00	-9.50	8.43
		CON+PLA	11.48	.15	-2.54	25.50
		CON+SW	6.11	.61	-6.86	19.07
	CON+PLA	RT+PLA	-12.01	.12	-26.03	2.01
		RT+SW	-11.48	.15	-25.50	2.54
		CON+SW	-5.37	.84	-22.23	11.49
	CON+SW	RT+PLA	-6.64	.54	-19.61	6.32
		RT+SW	-6.11	.61	-19.07	6.86
		CON+PLA	5.37	.84	-11.49	22.23
Knee flexion 180º/s	RT+PLA	RT+SW	.71	.99	-4.89	6.31
		CON+PLA	10.95*	.01	2.20	19.70
		CON+SW	9.47*	.02	1.37	17.56
	RT+SW	RT+PLA	-.71	.99	-6.31	4.89
		CON+PLA	10.24*	.02	1.48	18.99
		CON+SW	8.75*	.03	.66	16.85
	CON+PLA	RT+PLA	-10.95*	.01	-19.70	-2.20
		RT+SW	-10.24*	.02	-18.99	-1.48
		CON+SW	-1.48	.98	-12.01	9.04
	CON+SW	RT+PLA	-9.47*	.02	-17.56	-1.37
		RT+SW	-8.75*	.03	-16.85	-.66
		CON+PLA	1.48	.98	-9.04	12.01
Knee flexion 60º/s	RT+PLA	RT+SW	-4.86	.21	-11.32	1.61
		CON+PLA	6.84	.29	-3.27	16.95
		CON+SW	7.47	.16	-1.88	16.82
	RT+SW	RT+PLA	4.86	.21	-1.61	11.32
		CON+PLA	11.70*	.02	1.59	21.81
		CON+SW	12.33*	.00	2.98	21.68
	CON+PLA	RT+PLA	-6.84	.29	-16.95	3.27
		RT+SW	-11.70*	.02	-21.81	-1.59
		CON+SW	.63	1.00	-11.53	12.79
	CON+SW	RT+PLA	-7.47	.16	-16.82	1.88
		RT+SW	-12.33*	.00	-21.68	-2.98

		CON+PLA	-.63	1.00	-12.79	11.53
Elbow flexion 180º/s	RT+PLA	RT+SW	-.38	.99	-3.54	2.79
		CON+PLA	5.06*	.04	.11	10.00
		CON+SW	1.35	.87	-3.22	5.92
	RT+SW	RT+PLA	.38	.99	-2.79	3.54
		CON+PLA	5.43*	.03	.49	10.37
		CON+SW	1.72	.76	-2.85	6.30
	CON+PLA	RT+PLA	-5.06*	.04	-10.00	-.11
		RT+SW	-5.43*	.03	-10.37	-.49
		CON+SW	-3.71	.37	-9.65	2.24
	CON+SW	RT+PLA	-1.35	.87	-5.92	3.22
		RT+SW	-1.72	.76	-6.30	2.85
		CON+PLA	3.71	.37	-2.24	9.65
Elbow flexion 60º/s	RT+PLA	RT+SW	-.17	1.00	-3.19	2.84
		CON+PLA	5.48*	.02	.77	10.20
		CON+SW	2.09	.59	-2.27	6.45
	RT+SW	RT+PLA	.17	1.00	-2.84	3.19
		CON+PLA	5.66*	.01	.95	10.37
		CON+SW	2.26	.53	-2.09	6.62
	CON+PLA	RT+PLA	-5.48*	.02	-10.20	-.77
		RT+SW	-5.66*	.01	-10.37	-.95
		CON+SW	-3.40	.40	-9.06	2.27
	CON+SW	RT+PLA	-2.09	.59	-6.45	2.27
		RT+SW	-2.26	.53	-6.62	2.09
		CON+PLA	3.40	.40	-2.27	9.06

Sig. significance; RT+PLA: resistance training with placebo; RT+SW: resistance training with microfiltered seawater supplementation; CON+PLA: control with placebo; CON+SW: control with microfiltered seawater supplementation.

Supplementary Materials Table S3. Post-hoc test on bone markers.

Measure	Groups being compared		Mean Difference	Sig.	95% Confidence Interval	
					Lower Bound	Upper Bound
Global BMD	RT+PLA	RT+SW	-.04	.24	-.10	.02
		CON+PLA	-.03	.83	-.13	.07
		CON+SW	-.01	.99	-.10	.08
	RT+SW	RT+PLA	.04	.24	-.02	.10
		CON+PLA	.01	.99	-.09	.11
		CON+SW	.04	.72	-.05	.12
	CON+PLA	RT+PLA	.03	.83	-.07	.13
		RT+SW	-.01	.99	-.11	.09
		CON+SW	.02	.95	-.09	.14
	CON+SW	RT+PLA	.01	.99	-.08	.10
		RT+SW	-.04	.72	-.12	.05
		CON+PLA	-.02	.95	-.14	.09
Hip BMD	RT+PLA	RT+SW	-.04	.49	-.11	.03
		CON+PLA	-.03	.92	-.14	.09
		CON+SW	-.02	.93	-.13	.08
	RT+SW	RT+PLA	.04	.49	-.03	.11
		CON+PLA	.01	1.00	-.11	.13
		CON+SW	.01	.98	-.09	.12
	CON+PLA	RT+PLA	.03	.92	-.09	.14
		RT+SW	-.01	1.00	-.13	.11
		CON+SW	.00	1.00	-.13	.14
	CON+SW	RT+PLA	.02	.93	-.08	.13
		RT+SW	-.01	.98	-.12	.09
		CON+PLA	.00	1.00	-.14	.13
Spine BMD	RT+PLA	RT+SW	-.04	.61	-.12	.04
		CON+PLA	.01	.99	-.12	.15
		CON+SW	-.04	.75	-.16	.07
	RT+SW	RT+PLA	.04	.61	-.04	.12
		CON+PLA	.05	.73	-.08	.19
		CON+SW	-.01	1.00	-.12	.11
	CON+PLA	RT+PLA	-.01	.99	-.15	.12
		RT+SW	-.05	.73	-.19	.08
		CON+SW	-.06	.76	-.22	.10
	CON+SW	RT+PLA	.04	.75	-.07	.16
		RT+SW	.01	1.00	-.11	.12
		CON+PLA	.06	.76	-.10	.22
P1NP	RT+PLA	RT+SW	1.9	.88	-5.0	8.8
		CON+PLA	5.1	.65	-6.3	16.5
		CON+SW	.3	1.00	-9.7	10.4
	RT+SW	RT+PLA	-1.9	.88	-8.8	5.0
		CON+PLA	3.1	.89	-8.3	14.5
		CON+SW	-1.6	.97	-11.7	8.4

	CON+PLA	RT+PLA	-5.1	.65	-16.5	6.3
		RT+SW	-3.1	.89	-14.5	8.3
		CON+SW	-4.8	.79	-18.3	8.8
	CON+SW	RT+PLA	-.3	1.00	-10.4	9.7
		RT+SW	1.6	.97	-8.4	11.7
		CON+PLA	4.8	.79	-8.8	18.3
BCTX/1000	RT+PLA	RT+SW	.0	.98	-.1	.1
		CON+PLA	.0	.99	-.1	.1
		CON+SW	-.1	.33	-.2	.0
	RT+SW	RT+PLA	.0	.98	-.1	.1
		CON+PLA	.0	1.00	-.1	.1
		CON+SW	-.1	.21	-.2	.0
	CON+PLA	RT+PLA	.0	.99	-.1	.1
		RT+SW	.0	1.00	-.1	.1
		CON+SW	-.1	.45	-.2	.1
	CON+SW	RT+PLA	.1	.33	.0	.2
		RT+SW	.1	.21	.0	.2
		CON+PLA	.1	.45	-.1	.2

Sig. significance; RT+PLA: resistance training with placebo; RT+SW: resistance training with microfiltered seawater supplementation; CON+PLA: control with placebo; CON+SW: control with microfiltered seawater supplementation.

Supplementary Materials Table S4. Post-hoc test on body composition.

Measure	Groups being compared		Mean Difference	Sig.	95% Confidence Interval	
					Lower Bound	Upper Bound
Fat mass	RT+PLA	RT+SW	-1418.24	.77	-5274.32	2437.84
		CON+PLA	1040.15	.97	-4766.55	6846.86
		CON+SW	-4664.69	.14	-10261.02	931.65
	RT+SW	RT+PLA	1418.24	.77	-2437.84	5274.32
		CON+PLA	2458.39	.69	-3366.32	8283.11
		CON+SW	-3246.45	.43	-8861.46	2368.57
	CON+PLA	RT+PLA	-1040.15	.97	-6846.86	4766.55
		RT+SW	-2458.39	.69	-8283.11	3366.32
		CON+SW	-5704.84	.16	-12802.51	1392.82
	CON+SW	RT+PLA	4664.69	.14	-931.65	10261.02
		RT+SW	3246.45	.43	-2368.57	8861.46
		CON+PLA	5704.84	.16	-1392.82	12802.51
Muscle mass	RT+PLA	RT+SW	-451.05	.97	-3167.12	2265.01
		CON+PLA	1335.01	.83	-2754.99	5425.02
		CON+SW	357.99	1.00	-3583.83	4299.82
	RT+SW	RT+PLA	451.05	.97	-2265.01	3167.12
		CON+PLA	1786.06	.67	-2316.62	5888.75
		CON+SW	809.05	.95	-3145.94	4764.03
	CON+PLA	RT+PLA	-1335.01	.83	-5425.02	2754.99
		RT+SW	-1786.06	.67	-5888.75	2316.62
		CON+SW	-977.02	.96	-5976.32	4022.29
	CON+SW	RT+PLA	-357.99	1.00	-4299.82	3583.83
		RT+SW	-809.05	.95	-4764.03	3145.94
		CON+PLA	977.02	.96	-4022.29	5976.32
Fat percentage	RT+PLA	RT+SW	-.74	.90	-3.50	2.03
		CON+PLA	.99	.92	-3.17	5.15
		CON+SW	-3.51	.11	-7.52	.50
	RT+SW	RT+PLA	.74	.90	-2.03	3.50
		CON+PLA	1.73	.70	-2.45	5.90
		CON+SW	-2.77	.28	-6.80	1.25
	CON+PLA	RT+PLA	-.99	.92	-5.15	3.17
		RT+SW	-1.73	.70	-5.90	2.45
		CON+SW	-4.50	.10	-9.59	.59
	CON+SW	RT+PLA	3.51	.11	-.50	7.52
		RT+SW	2.77	.28	-1.25	6.80
		CON+PLA	4.50	.10	-.59	9.59

Sig. significance; RT+PLA: resistance training with placebo; RT+SW: resistance training with microfiltered seawater supplementation; CON+PLA: control with placebo; CON+SW: control with microfiltered seawater supplementation.

Supplementary Materials Table S5. Post-hoc test on SF-36.

Measure	Groups being compared		Mean Difference	Sig.	95% Confidence Interval	
					Lower Bound	Upper Bound
General health	RT+PLA	RT+SW	-3.94	.58	-12.06	4.17
		CON+PLA	-3.42	.88	-15.59	8.75
		CON+SW	-1.08	1.00	-12.81	10.65
	RT+SW	RT+PLA	3.94	.58	-4.17	12.06
		CON+PLA	.52	1.00	-11.64	12.69
		CON+SW	2.87	.92	-8.86	14.60
	CON+PLA	RT+PLA	3.42	.88	-8.75	15.59
		RT+SW	-.52	1.00	-12.69	11.64
		CON+SW	2.34	.98	-12.48	17.17
	CON+SW	RT+PLA	1.08	1.00	-10.65	12.81
		RT+SW	-2.87	.92	-14.60	8.86
		CON+PLA	-2.34	.98	-17.17	12.48
Physical functioning	RT+PLA	RT+SW	.66	1.00	-9.85	11.17
		CON+PLA	6.76	.68	-9.00	22.52
		CON+SW	6.30	.70	-8.90	21.49
	RT+SW	RT+PLA	-.66	1.00	-11.17	9.85
		CON+PLA	6.10	.74	-9.67	21.86
		CON+SW	5.63	.77	-9.56	20.83
	CON+PLA	RT+PLA	-6.76	.68	-22.52	9.00
		RT+SW	-6.10	.74	-21.86	9.67
		CON+SW	-.46	1.00	-19.67	18.74
	CON+SW	RT+PLA	-6.30	.70	-21.49	8.90
		RT+SW	-5.63	.77	-20.83	9.56
		CON+PLA	.46	1.00	-18.74	19.67
Physical role	RT+PLA	RT+SW	-4.03	.69	-13.57	5.50
		CON+PLA	-2.47	.97	-16.78	11.83
		CON+SW	3.27	.93	-10.52	17.06
	RT+SW	RT+PLA	4.03	.69	-5.50	13.57
		CON+PLA	1.56	.99	-12.74	15.86
		CON+SW	7.30	.51	-6.49	21.09
	CON+PLA	RT+PLA	2.47	.97	-11.83	16.78
		RT+SW	-1.56	.99	-15.86	12.74
		CON+SW	5.74	.82	-11.69	23.17
	CON+SW	RT+PLA	-3.27	.93	-17.06	10.52
		RT+SW	-7.30	.51	-21.09	6.49
		CON+PLA	-5.74	.82	-23.17	11.69
Bodily pain	RT+PLA	RT+SW	-7.30	.31	-18.35	3.76
		CON+PLA	5.17	.85	-11.41	21.75
		CON+SW	-3.06	.96	-19.04	12.93
	RT+SW	RT+PLA	7.30	.31	-3.76	18.35
		CON+PLA	12.47	.21	-4.11	29.04
		CON+SW	4.24	.90	-11.74	20.22

	CON+PLA	RT+PLA	-5.17	.85	-21.75	11.41
		RT+SW	-12.47	.21	-29.04	4.11
		CON+SW	-8.22	.71	-28.43	11.98
	CON+SW	RT+PLA	3.06	.96	-12.93	19.04
		RT+SW	-4.24	.90	-20.22	11.74
		CON+PLA	8.22	.71	-11.98	28.43
Emotional role	RT+PLA	RT+SW	-3.63	.56	-10.88	3.62
		CON+PLA	-2.61	.92	-13.48	8.26
		CON+SW	-.63	1.00	-11.11	9.85
	RT+SW	RT+PLA	3.63	.56	-3.62	10.88
		CON+PLA	1.02	.99	-9.86	11.89
		CON+SW	3.00	.88	-7.49	13.48
	CON+PLA	RT+PLA	2.61	.92	-8.26	13.48
		RT+SW	-1.02	.99	-11.89	9.86
		CON+SW	1.98	.98	-11.27	15.23
	CON+SW	RT+PLA	.63	1.00	-9.85	11.11
		RT+SW	-3.00	.88	-13.48	7.49
		CON+PLA	-1.98	.98	-15.23	11.27
Social function	RT+PLA	RT+SW	-5.08	.39	-13.42	3.25
		CON+PLA	-9.99	.16	-22.50	2.52
		CON+SW	-5.30	.66	-17.36	6.76
	RT+SW	RT+PLA	5.08	.39	-3.25	13.42
		CON+PLA	-4.91	.73	-17.42	7.60
		CON+SW	-.22	1.00	-12.27	11.84
	CON+PLA	RT+PLA	9.99	.16	-2.52	22.50
		RT+SW	4.91	.73	-7.60	17.42
		CON+SW	4.69	.85	-10.55	19.93
	CON+SW	RT+PLA	5.30	.66	-6.76	17.36
		RT+SW	.22	1.00	-11.84	12.27
		CON+PLA	-4.69	.85	-19.93	10.55
Vitality	RT+PLA	RT+SW	-1.69	.97	-11.38	7.99
		CON+PLA	4.10	.88	-10.43	18.63
		CON+SW	2.88	.95	-11.13	16.88
	RT+SW	RT+PLA	1.69	.97	-7.99	11.38
		CON+PLA	5.79	.72	-8.74	20.32
		CON+SW	4.57	.83	-9.43	18.58
	CON+PLA	RT+PLA	-4.10	.88	-18.63	10.43
		RT+SW	-5.79	.72	-20.32	8.74
		CON+SW	-1.22	1.00	-18.93	16.48
	CON+SW	RT+PLA	-2.88	.95	-16.88	11.13
		RT+SW	-4.57	.83	-18.58	9.43
		CON+PLA	1.22	1.00	-16.48	18.93
Mental health	RT+PLA	RT+SW	-4.65	.53	-13.66	4.36
		CON+PLA	-3.20	.93	-16.71	10.31
		CON+SW	-4.55	.80	-17.57	8.47
	RT+SW	RT+PLA	4.65	.53	-4.36	13.66
		CON+PLA	1.45	.99	-12.06	14.96

	CON+SW	.10	1.00	-12.92	13.12
CON+PLA	RT+PLA	3.20	.93	-10.31	16.71
	RT+SW	-1.45	.99	-14.96	12.06
	CON+SW	-1.35	1.00	-17.81	15.11
CON+SW	RT+PLA	4.55	.80	-8.47	17.57
	RT+SW	-.10	1.00	-13.12	12.92
	CON+PLA	1.35	1.00	-15.11	17.81

Sig. significance; RT+PLA: resistance training with placebo; RT+SW: resistance training with microfiltered seawater supplementation; CON+PLA: control with placebo; CON+SW: control with microfiltered seawater supplementation.
